# Malaria was a weak selective force in ancient Europeans

**DOI:** 10.1038/s41598-017-01534-5

**Published:** 2017-05-03

**Authors:** Pere Gelabert, Iñigo Olalde, Toni de-Dios, Sergi Civit, Carles Lalueza-Fox

**Affiliations:** 1 Institute of Evolutionary Biology (CSIC-Universitat Pompeu Fabra), 08003 Barcelona, Spain; 2 Department of Statistics, Faculty of Biology, University of Barcelona, 08028 Barcelona, Spain

## Abstract

Malaria, caused by *Plasmodium* parasites, is thought to be one of the strongest selective forces that has shaped the genome of modern humans and was endemic in Europe until recent times. Due to its eradication around mid-twentieth century, the potential selective history of malaria in European populations is largely unknown. Here, we screen 224 ancient European genomes from the Upper Palaeolithic to the post-Roman period for 22 malaria-resistant alleles in twelve genes described in the literature. None of the most specific mutations for malaria resistance, like those at *G6PD*, *HBB* or Duffy blood group, have been detected among the available samples, while many other malaria-resistant alleles existed well before the advent of agriculture. We detected statistically significant differences between ancient and modern populations for the *ATP2B4*, *FCGR2B* and *ABO* genes and we found evidence of selection at *IL-10* and *ATP2B4* genes. However it is unclear whether malaria is the causative agent, because these genes are also involved in other immunological challenges. These results suggest that the selective force represented by malaria was relatively weak in Europe, a fact that could be associated to a recent historical introduction of the severe malaria pathogen.

## Introduction

The malaria parasite *Plasmodium falciparum* is one of the main causes of child mortality worldwide, although other species from the same genus, *P. vivax, P. malariae* and *P. ovale* are also causative agents of the disease. Therefore, it is not surprising that malaria is one of the strongest known selective pressures that have recently shaped the human genome. Malaria is the evolutionary driving force behind sickle-cell disease (HbS), thalassemia, glucose-6-phosphatase deficiency (G6PD) and other erythrocyte defects that together comprise the most common Mendelian diseases of humankind. Remarkably, populations from different geographical areas have developed different genetic mechanisms adapted to malaria resistance; for instance, the HbS allele at the *HBB* gene is common in Africa but rare in Southeast Asia, whereas the HbE alelle shows a reversed pattern. Resistance to malaria includes primarily genes involved in immunological response, but also some involved in inflammation and cell adhesion and even genes related to metabolic pathways. The fact that different malaria-resistance alleles have arisen in different places suggests that this adaptation occurred relatively recent in human history, at least well after the Out of Africa migration^1^.

*P. falciparum* may have rapidly spread from its African tropical origins to other tropical and subtropical regions of the world only within the last 6,000 years^2^. The recent origin of the worldwide *P. falciparum* populations, which are the most malignant of human malarial parasites, may account for its virulence^3^. Thus, haplotype analysis at the G6PD locus suggests that the African resistance allele to *P. falciparum* originated within the last 10,000 years^4^ while a similar analysis at the HbE alleles (variants of the *HBB* gene) in Southeast Asia yielded an even more recent date, around 5,000 years ago^5^. These and other estimates associate the resistance to *P. falciparum* with the onset of agriculture and the emergence of favourable environments for mosquito proliferation after the clearance of woodlands for farming. Falciparum-like illness (I.e. miasmas) appears to be very recent in Europe. It seems to have spread from India to the West, appearing in ancient Greece only by the 4th century BCE, where it was implicated in the decline of many city-state populations^2^. The retrieval of the eradicated European *P. falciparum* from 70-year-old slides from a malaria-endemic region in Spain confirms a recent, phylogenetic link with present-day Indian haplotypes^6^. Therefore, it seems unlikely that *P. falciparum* would have had a significant impact on prehistoric European genomes.

On the other hand, *P. vivax* (and probably *P. ovale* and *P. malaria*) is likely an ancient parasite that evolved in Africa and spread to the rest of the world with the Out of Africa migration, around 60,000 years ago^2^. Phylogenetic analyses suggest that *P. vivax* could have an African origin but has largely disappeared from this continent after the spread of Duffy negative resistance^7^. The biology of *P. vivax* that makes it highly adapted to maintain itself in small mobile groups of hosts and its mild effect also supports the idea that it is an ancient human parasite. The limited data available does show that *P. vivax* and *P. falciparum* may have had at least partially different effects on the human genome^1^.

Malaria was endemic in Europe until very recently. Historical data describe malaria as affecting people as north as England, Scandinavia or Russia^2^. However, while there is information of some malaria-resistance alleles from Europe, notably from the Mediterranean area (such as the *HBB* and the *G6PD* variants), little is known about the magnitude of selection due to malaria in the continent because of the eradication of the parasite around 1950. The recent retrieval of more than 250 ancient genomes from different moments of the prehistory of Europe^8–11^ and the publication of several Genome wide association studies (GWAS) on malaria^12, 13^ now allows for a genome-wide screening of malaria-resistance alleles along space and time. Using this approach, we aim to understand the role of this disease as a selective force in the shaping of the gene pool of modern Europeans.

## Results

### Sample size and allele imputation

**Figure 1:**
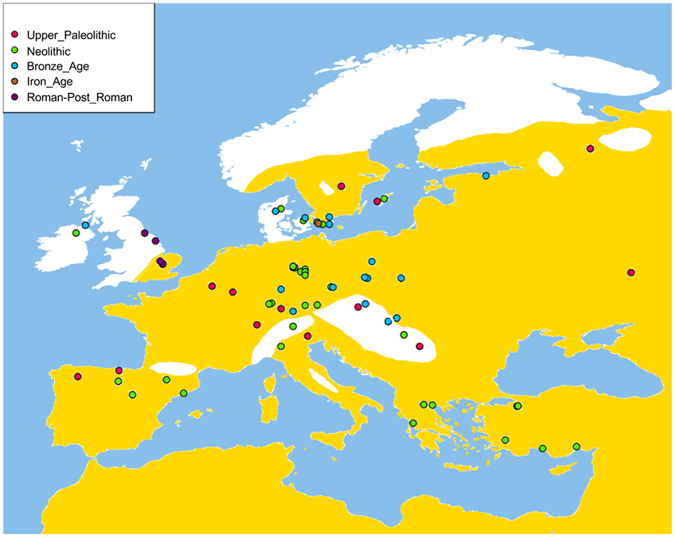
Geographical distribution of ancient European samples included in the analysis of the malaria-resistant mutations. The map depicts the malaria-endemic zone^57^. The map was made using Worldmap package 1.3.1 of R software 3.2.2 (http://cran.r-project.org), and modified with Gimp 2.6 (https://www.gimp.org) and Pinta 1.6 (https://pinta-project.com).

It was possible to screen 224 ancient individuals (Fig. 1 and Table S1) for 20 single nucleotide polymorphisms (SNPs) and two single nucleotide-deletions described to play a role in malaria resistance and distributed in twelve different genes: *G6PD*, *HBB*, *ACKRQ*, *FCGR2B*, *TIRAP*, *ATP2B4*, *GRK5*, *IL-10*, *MARVELD3*, *CD36*, *CD40LG* and *ABO* blood group system (Table 1). Due to low coverage on many ancient samples, complete genotypes are typically not available for all loci; therefore, we needed to rely on imputation methods to infer genotypes, using the 1,000 Genomes phased samples as a reference panel and Beagle-4 software^14^.

**Table 1:** Summary of the genetic variants analysed in the study and the hypothetical functional explanation in relation to malaria resistance, when available.

Reference SNP ID	Gene	Relation to malaria resistance	World distribution (% of resistant variant)	SNPs recovered from genome wide data samples (2 N)	SNPs recovered from targeted capture data (N)
rs1050828	*G6PD*	G6PD A−, mutation of a Val to a Met in the residue 68 that causes instability	Europe: 0; Africa: 13; America: 1; Asia: 0	79,35%	46,01%
rs1050829	*G6PD*	G6PD A+ produces a substitution of a Asn to a Asp in the residue 126	Europe: 0; Africa: 34; America: 3; Asia: 0	79,35%	35,58%
rs5030868	*G6PD*	G6PD Mediterranean, change from a Ser to a Phe in position 188 causing abnormality	Europe: 0; Africa: 0; America: 0; Asia: 0	23,91%	3,067%
rs137852314	*G6PD*	Mutation associated cause in enzyme derangement and associated with AluI site	Europe: 0; Africa: 0; America: 0; Asia: 0	23,91%	3,067%
rs76723693	*G6PD*	Substitution found in subjects with G6PD A− phenotype	Europe: 0; Africa: 1; America: 0; Asia: 0	80,34%	1,84%
rs137852328	*G6PD*	Substitution found in subjects with G6PD A− phenotype	Europe: 0; Africa: 0; America: 0; Asia: 0	26,087%	1,84%
rs372091	*HBB*	Identified in a GWAS study	Europe: 0; Africa: 7; America: 0; Asia: 0	93,48%	41,72%
rs33930165	*HBB*	Modification of erythrocyte structure, hemoglobine C	Europe: 0; Africa: 1; America: 0; Asia: 0	96,74%	1,84%
rs33950507	*HBB*	Modification of erythrocyte structure, hemoglobine E	Europe: 0; Africa: 0; America: 0; Asia: 1	96,74%	1,84%
rs334	*HBB*	Modification of erythrocyte structure, hemoglobine S	Europe: 0; Africa: 10; America: 1; Asia: 0	76,087%	1,84%
rs2814778	*ACKR1*	Change in the structure of erytrochyte surface protein	Europe: 1; Africa: 96; America: 8; Asia: 0	93,48%	40,91%
rs1050501	*FCGR2B*	Enhancement of phacocytosis	Europe: 14; Africa: 25;America: 9; Asia: 20	53,26%	2,45%
rs8177374	*TIRAP*	Substitution of a Ser to a Leu in the residue 180 attenuating TLR2 signal transduction	Europe: 19; Africa: 1; America: 8; Asia: 9	59,78%	3,68%
rs4951074	*ATP2B4*	Mutation in a Ca2+ pump in the erythrocyte affecting the P falciparum cycle	Europe: 10; Africa: 37; America: 6; Asia: 7	95,65%	1,84%
rs10900585	*ATP2B4*	Mutation in a Ca2+ pump in the erythrocyte affecting the P falciparum cycle	Europe: 90; Africa: 58; America: 93; Asia: 92	90,22%	26,99%
rs2230345	*GRK5*	Ser/Thr Kinase mutation that makes difficult the internalization of P falciparum in erythrocyte	Europe: 1; Africa: 30; America: 3; Asia: 5	72,83%	6,13%
rs1800890	*IL-10*	*IL-10* mutations decreases the level of this cytokines in the plasma	Europe: 37; Africa: 20; America: 24; Asia: 12	35,87%	1,84%
rs2334880	*MARVELD3*	Identified in a GWAS study	Europe: 84; Africa: 60; America: 90; Asia: 98	85,87%	11,65%
rs8176719	*ABO*	Individuals homozygous classified as group O, associated with decreased risk of malaria.	Europe: 61; Africa: 71; America: 77; Asia:61	45,65%	0%
rs8176746	*ABO*	Determines the production of B antigens, associated with increased risk of malaria	Europe: 92; Africa: 83; America: 95; Asia:79	75%	31,29%
rs3092945	*CD40LG*	Mutation in a glycoprotein involved in B cell proliferation, antigen presenting cell activation is important in the immune response to infection	Europe: 1; Africa: 31; America: 4; Asia: 0	81,52%	0%
rs201346212	*CD36*	Scavenger receptor CD36 plays important roles in malaria, including the sequestration of parasite-infected erythrocytes in microvascular capillaries	Europe: 0; Africa: 1; America: 0; Asia: 0	81,52%	0%

The allelic frequencies have been estimated from data from the 1,000 Genomes. The recovery statistics are also presented in this Table.

After imputation, the number of genotypes recovered for each nucleotide position was variable (Table 1 and Table S1). The proportion of recovered haplotypes in whole genome sequence data fluctuated from a maximum 96.74% in *HBB* SNP: rs33950507 and rs334 to a minimum 23.91% in *ATP2B4* SNP rs5030868. The proportion of recovered alleles in targeted capture data samples fluctuated from a maximum of 40.91% in *ACKRQ* SNP: rs2814778 to zero observations for *CD36* SNP: rs3092945, *CD40LG* SNP: rs201346212, and *ABO* SNP: rs817671. A dataset with genotypes from 92 genome-wide samples is presented in Table S2.

Even with such data limitations, we were able to detect a substantial number of carriers of the proposed malaria resistance alleles in our ancient genome dataset (Table 2). It is noteworthy that different derived genetic variants (at *TIRAP*, *ATP2B4*, *MARVELD3, ABO*, and *IL-10*) were already present prior to the arrival of farming, in Upper Palaeolithic and/or Mesolithic individuals. For other loci, the derived alleles are only seen in very recent periods. For instance, only one of the Iron Age individuals carries the T allele (rs2230345) at *GRK5* gene, one individual carries the T allele (rs8176746) at *ABO* gene and only two individuals (one Bronze Age Russian and one Post-Roman British) carry the C allele (rs1050501) at the *FCGR2B* gene.

**Table 2:** Frequencies of malaria-resistant alleles by time period in nine selected genes and 224 available ancient European genomes.

	FCGR2B	TIRAP	GRK5	IL-10	ATP2B4		MARVELD3	ABO	
	rs1050501	rs8177374	rs2230345	rs1800890	rs4951074	rs10900585	rs2334880	rs8176746	rs8176719
UPPER PALEOLITHIC	TT = 7	CC = 6 CT = 3	AA = 10 A* = 1	AA = 1 TA = 2 TT = 1	GG = 12 GA = 2	TT = 12 GT = 2	GG = 5 AG = 9 G* = 7	GG = 8 G* = 9	– = 3 -G = 2 GG = 1
EARLY NEOLITHIC	TT = 10 T* = 1	CC = 3 CT = 8 TT = 1 T* = 2	AA = 13 A* = 2	AA = 2 TA = 2 TT = 1	GG = 17 GA = 1	TT = 13 T* = 2	GA = 1 GG = 16 A* = 3	GG = 24 G* = 12	– = 2 GG = 3 -G = 3
MIDDLE NEOLITHIC	TT = 1 T* = 1	TT = 1 C* = 2	AA = 3 A* = 2	TA = 1	GG = 5 GA = 1	TT = 4 GT = 2 T* = 4 G* = 1	GG = 4 G* = 2 A* = 1	GG = 4 G* = 8	– = 2
LATE NEOLITHIC	TT = 3 T* = 1	CC = 2 TT = 1 C* = 1	AA = 5 A* = 2	TA = 1	GG = 6	TT = 5 G* = 4 T* = 8	GG = 6 G* = 1	GG = 5 G* = 14 T* = 1	– = 2 GG = 1 -G = 2
BRONZE AGE	TT = 24 TC = 1	CC = 15 CT = 3 TT = 1 C* = 1	AA = 19 A* = 2	AA = 1 TA = 4 TT = 4	GG = 20 GA = 4	TT = 23 GT = 1 T* = 10 G* = 5	GG = 16 GA = 5 AA = 1	GG = 21 GT = 1 G* = 6	–* = 4 GG = 4
IRON AGE	TT = 7	CC = 5	AA = 4 AT = 1	AA = 2, TT = 3	GG = 6 GA = 1	TT = 5 G* = 1,T* = 1	GG = 6 G* = 1 A* = 1	GG = 6 G* = 1	– = 3 GG = 1 -G = 1
ROMAN	TT = 2	CC = 2 CT = 1	AA = 5	TT = 3	GG = 5	TT = 5	GG = 5	GG = 5	– = 1
POST-ROMAN	TT = 2 CT = 1	CC = 2 CT = 4	AA = 6	TA = 3 TT = 2	GG = 8	TT = 8	GG = 7 GA = 1	GG = 8	– = 1 GG = 2 -G = 2
ANCIENT POPULATIONS	T = 98% C = 2%	C = 76% T = 24%	A = 99% T = 1%	C = 40%, T = 60%	G = 95% A = 5%	G = 8% T = 92%	G = 90% A = 10%	G = 99% T = 1%	- = 57,5% G = 42,5%
GLOBAL EUROPEAN	EUR:T = 86%,T = 14%	EUR:C = 81%,T = 19%	EUR:A = 99%,T = 1%	EUR:A = 63%,T = 37%	EUR:G = 90%,A = 10%	EUR:T = 90%,G = 10%	EUR:G = 84%,A = 16%	EUR:G = 92%,T = 8%	EUR:- = 61%, G = 39%

The frequencies of current European populations are displayed below the ancient populations. All genotypes included passed an imputation filter > 0.95. The * sign denotes that a specific allele is present, but we cannot confidently imputate the genotype. Only those SNPs showing at least one diverse allele have been included.

### Ancient-modern population comparisons

**Figure 2:**
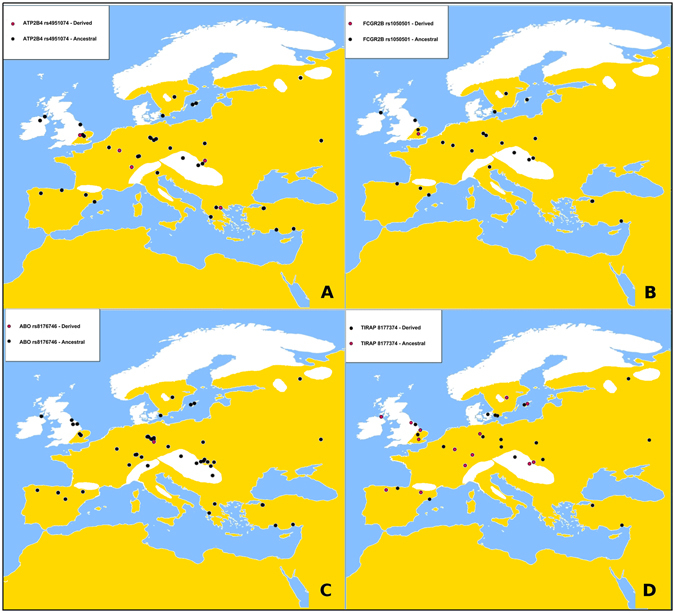
Geographical distribution of the rs8176746, rs4951074, rs1050501 and rs8177374 alleles in ancient European populations. The black circles represent those individuals with ancestral variants and red circles represent those individuals with derived variants presumably associated to malaria resistance. The map depicts the malaria-endemic zone^57^. The maps were made using Worldmap package 1.3.1 of R software 3.2.2 (http://cran.r-project.org), and modified with Gimp 2.6 (http://www.gimp.org) and Pinta 1.6 (http://pinta-project.com).

For three genetic variants, we observed significant frequency differences between ancient and modern European populations from the 1,000 Genomes project, after adjusting for multiple tests: rs1050501 (*FCGR2B*) (OR = 7.86; CI = 2.07–66.66; p = 2.2e-16), rs4951074 (*ATP2B4*) (OR = 2.16; CI = 1.06–4.96; p = 0.02) and rs8176746 (*ABO*) (OR = 9.63; CI = 2.54–81.49; p = 1.19e-5). The three genes show lower frequencies than expected of the derived alleles in ancient populations as compared to present-day Europeans. Samples with data from these three statistically significant ancient *vs* modern variants are not geographically clustered, as can be seen in Fig. 2. Therefore, no clear climatic or latitudinal pattern that could be associated to the malaria distribution emerges.

### Population structure

Although malaria was prevalent along the continent, from Russia and Scandinavia to Southern Europe, some resistance mutations such as *G6PD* B- are nowadays restricted to the Mediterranean, which suggests that the impact of the disease may have been different between European regions. We therefore tested for population structure at these loci.

The only SNP to show significant North-South differences in allele frequencies in modern Europeans was the rs8177374 SNP at *TIRAP* gene. The allelic frequency of the derived allele in South-Europe populations (IBS and TSI) was 34%, statistically significant higher than the 16% of the derived alleles in North-European populations (CEU, GBR and FIN) (codominant model: OR = 4.04; CI = 1.37–11.91; p = 1.428e-05 and log-additive model: OR = 2.20; CI = 1.57–3.08; p = 2.522e-06). This trend cannot be discerned in the ancient samples (see Fig. 2).

Although the problems of assessing population structure with a dataset of low-coverage, geographically and temporally disparate ancient genomes cannot be overlooked we sought to explore the genetic structure among ancient and modern European with an F_ST_ and G-test approach. We have grouped the ancient samples in eight periods (Upper Palaeolithic, Early Neolithic, Middle Neolithic, Late Neolithic, Bronze Age, Iron Age, Roman Age and Post-Roman Age) (Table S1). The F_ST_ pairwise comparisons did not reveal any structural differentiation in the established sub-groupings (Table S4). A G-test analysis detected a significant geographic differentiation of the variants associated with malaria resistance among the five present-day European populations (CEU, IBS, TSI, GBR, FIN) of the 1,000 Genomes database (G-statistic; 102.304, p-value: 0.01) (Table S5). This suggests that very fine differences in allele frequencies among these populations likely exist. We further explored this issue with Chi-squared test comparisons for each SNP between all pairs of populations; we found that all pairwise tests including Finns (FIN) are statistically significant, and also one North-South test (IBS *vs* CEU).

### Evidence of selection

**Figure 3:**
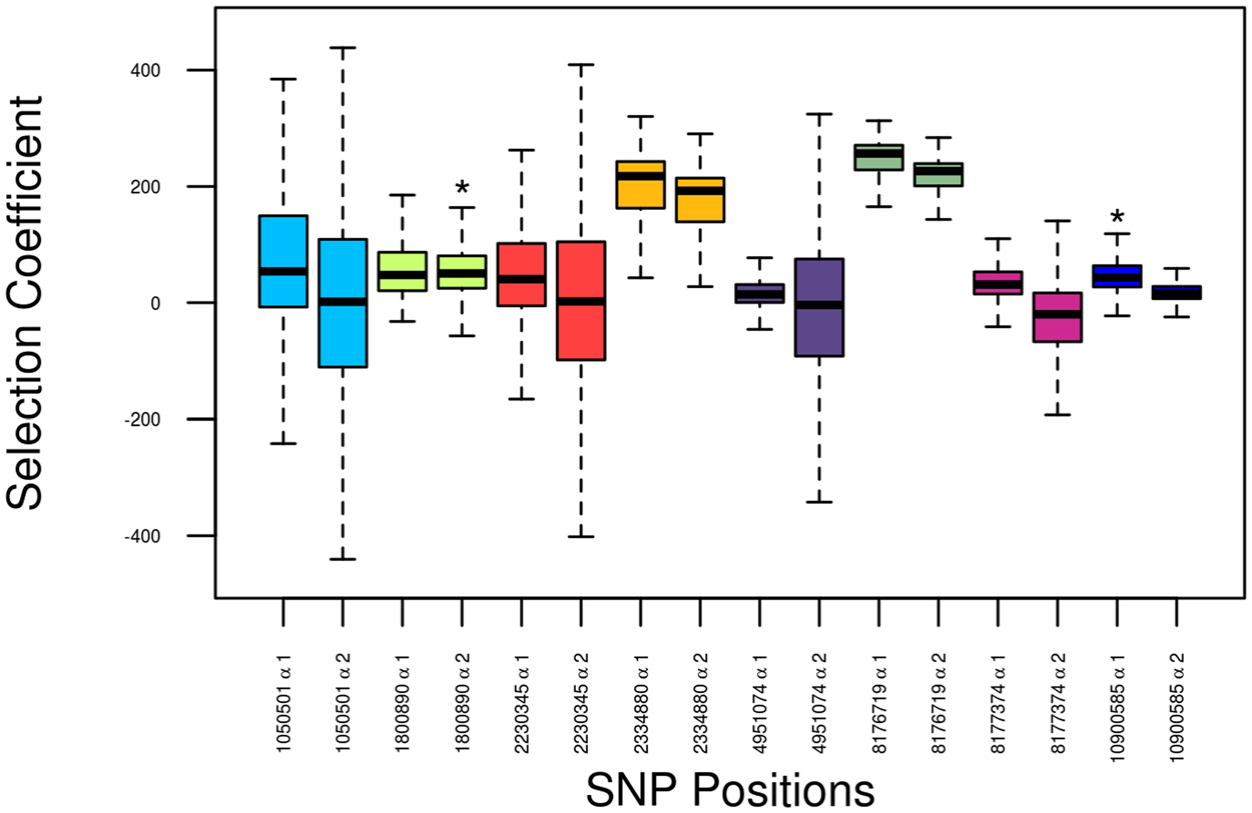
Mean values and standard deviations of the alpha 1 and alpha 2 selection coefficients for the malaria-resistant SNPs that exhibit variability (https://github.com/Schraiber/selection). *Statistically significant selection coefficients.

To further explore the possibility that changes in our time series data could be attributed to selection acting on the derived alleles, we used a Bayesian method that estimates the frequency trajectory of the alleles based on observed frequencies and sampling dates^15^. The “Selection” software estimates a posterior probability of selection coefficients for heterozygous haplotypes (alpha 1) and for homozygous derived haplotypes (alpha 2) for each SNP and also the allelic age. The mean values of the selection coefficients and the likelihood values are shown in Fig. 3, and the posterior distribution of selection coefficients (alpha1 and alpha2) are shown in Supplementary Figures S6–S13.

Two genetic variants showed statistically significant evidence of positive selection on the derived allele: rs1800890 SNP at *IL-10* gene with a favoured derived-homozygous genotype (alpha 2: mean value = 57.7; p = 0.046) and rs10900585 SNP at *ATP2B4* gene, with a favoured heterozygous genotype (alpha 1: mean value = 48.85; p = 0.015); p is the probability of the selection coefficient to be <0.

The allelic ages estimates showed that the oldest derived allele is rs8176719 at the *ABO* blood group gene. The age estimates of all alleles (Supplementary Table S6) predate our sampling period with the exception of rs1050501 at the *FCGR2B* gene that yielded an age of 9,500 years.

## Discussion

Globally, the comparison of the allele frequencies between ancient and current European populations did not reveal strong variations in most of the analysed genes. In most cases, and despite limitations in sample size, the allele frequencies in the ancient samples were very similar to those of the modern populations (Table 2).

The fact that we can, in most cases, detect the derived allele in a dataset based on a handful of ancient genomes implies that these alleles had already reached relatively high frequencies in the ancient populations. Even with the uncertainties associated to the small sample size, we provide for first time information about the ages of the derived malaria resistance alleles. With the single exception of the *FCGR2B* gene, all of them predate the emergence of the Neolithic by thousands of years. However, the period when they became prevalent and potentially important from a phenotypical point of view, can be much more recent. It has been suggested that the spread of malaria was triggered by the deforestation associated with the farming expansion^16^. However, our results do not indicate any clear shift in the allelic frequencies of the malaria-resistant genes in response to the onset of farming.

*P. vivax* is thought to be a relatively ancient parasite that probably was common when *Homo sapiens* colonized Europe, about 45,000 years ago^2^. It is therefore not surprising that this parasite could have had a significant impact on pre-Neolithic European as well as African genomes. The three traits that have been clearly shown by functional studies to protect against *P. vivax* are: Duffy negativity (*ACKR1* gene), Ovalocytosis (in South East Asian groups) and *G6PD* deficiency^17–19^. The first two have little if any effect on *P. falciparum* while *G6PD* seems to have more effect on *P. vivax* than on *P. falciparum*. So far, none of the ancient genomes available displays any mutation at the crucial *G6PD* or Duffy loci. In addition, we do not find a single carrier of the *HBB* mutation. We don’t know if this is attributable to the rather limited sample size or if their absence reflects a relatively recent origin of these mutations among European populations. The frequencies of these mutations are very low in current populations (for instance, there is not a single carrier among the European populations of the 1,000 Genomes project). However, the sample size for some of the SNPs, such as the rs372091 at the *HBB* locus, is not that small and currently includes 155 ancient genomes.

The current shortage of ancient genomic data from the Mediterranean area (where severe malaria was most prevalent in historic times) represents a limitation of our survey. This is partially attributable to the warm environmental conditions in the area, which are unfavourable to DNA preservation^20^. At present, only one complete genome (a Cardial Early Neolithic specimen)^21^ and several Neolithic individuals from the oriental Mediterranean peninsulas (Greece and Anatolia)^22, 23^ derive from the thermo-Mediterranean zone^21^. However, a significant number of samples derive from regions that are close to the Mediterranean shores (Fig. 1).

While looking at the variation among modern Europeans with the G-statistic, the present-day frequencies of the SNPs related with malaria resistance denote the presence of substructure that should be further investigated with additional modern populations and neutral variation SNPs. The structure could be partly influenced by the distinctiveness of the Finnish sample but also by the SNP at *TIRAP* gene (rs8177374) (Table S5) that shows a clear latitudinal trend confirmed with the OR results. However, the same mutation has also been related to resistance to bacterial infections such as tuberculosis^24^. Therefore, it is unclear if the clinal pattern of variation for this allele is in fact associated to malaria infection or to other selective forces acting on a latitudinal basis.

Three genes (*FCGR2B*, *ATP2B4* and *ABO*) show differences in the allelic frequency between ancient and modern Europeans. This could in principle be the signal of recent events of positive selection acting upon the associated SNPs.

The mutation at the *FCGR2B* gene (an ILE > THR change in the 232 residue of the protein) has been proposed to have a strong protective effect against severe malaria (OR = 0.56, p < 0.001) in African populations due to the enhancement of phagocythosis of *P. falciparum* infected-human erythrocytes^25^. This could explain the high frequency of this mutation in malaria endemic areas, although the same mutation has also been described as causative of Systemic Lupus Erythematosus^25, 26^. The only two individuals that carry this mutation are from periods and regions where malaria would be endemic in historical times: one is a Bronze Age individual from southern Russia and the other one an Anglo-Saxon from southern England. However, some older specimens (Table S1) also seem to carry the mutation, although the imputation test in those cases did not meet the 0.95 threshold. Polymorphisms at *ATP2B4* gene, encoding for membrane calcium-transporter protect against malaria because of its function in homoeostasis control of the erythrocyte membrane^27^, although this association needs to be assessed with further functional evidence. The rs8176746 SNP at the *ABO* gene is in linkage disequilibrium with rs8176719 SNP that determines B antigens. The derived allele has been associated with increased risk of severe malaria^28^.

To evaluate if these genes could bear the footprints of recent positive selection (as opposed to neutral processes such as drift or migration), we consulted a genome browser with information about such signatures^29^, but none of them displayed any evidence of selective sweeps. An alternative to selection would be migration; it has been recently demonstrated that European populations were modelled by three genetic components that overlapped in the last thousands of years: the Mesolithic hunter-gatherers, the Early Neolithic farmers and the Late Neolithic steppe migrants^11, 30^. Although the right approach to distinguish between selection and migration would be the one followed by Mathieson *et al*.^9^, the lack of data in the original source populations for most of our SNPs precludes this approach. However, we noticed that differences in allelic frequencies detected in *FCGR2B* and *ATP2B4* are restricted to very recent periods -after the Bronze Age- when the general composition of the European populations was already established. Therefore, the trend observed towards the increase of the derived allele is unlikely to be attributed to these ancient migrations.

We have modelled all the SNPs with an approach that rejects neutrality across a time series and found evidence for selection in SNPs rs10900585 at *ATP2B4* and rs1800890 at *IL-10* genes. This suggests that selection acted on these genes, an evidence that should be further explored from a functional point of view. The fact that no previous evidence of selective sweeps on these genes has been reported could be explained if the timing of the selective event was very recent or if the effect was regionally restricted. Nevertheless, it is reasonable to discard large-scale and dramatic selective events such as those seen for the lactase gene^9^.

In conclusion, it seems that most of the screened alleles were equally prevalent in past than in present time; in those alleles were differences are statistically significant, we cannot discard the effect of drift or other selective forces acting upon these immunity genes. This could indicate a weak adaptation to malaria, due to either the benignity of symptoms caused by *P. vivax* or to an undetectable adaptive response related to a very recent arrival to the European continent (which seems to be the case of severe malaria caused by *P. falciparum*). We have shown however that, despite the large geographic distribution, malaria was likely not an important selective pressure in ancient European populations and not nearly as important as it is in Africa today. Additionally we demonstrate that the study of the selective effect of malaria on ancient genomes is a feasible approach that will likely provide more information in the future with increasing sample sizes, especially from the Mediterranean area. With additional genomic data, and also the retrieval of DNA from the pathogen directly from ancient bones^31^, it would be possible to check if local selective events took place and to understand the role of malaria in the shaping of modern European genomes.

## Methods

### Ancient genomes

We have analysed all ancient European -*sensu lato*- genomes with a mean sequence coverage >1x published so far^11, 21–23, 32–39^. We have also included those genomes for which only targeted capture data is available^9, 10, 30, 40–42^. Our final dataset comprises genome-wide-data from 224 individuals (Fig. 1,Table S1) from which we have at least information for one of the malaria-resistant markers.

### Screened genetic variants

We have screened all malaria-resistant alleles from the literature, even if most of them have been described as being selective in current African populations and involved in *P. falciparum* resistance. Of course this cannot be directly extrapolated to European populations but the limited information on the selective effect of *P. vivax* or *P. falciparum* on Europeans -especially outside the Mediterranean area- makes alternative approaches unfeasible. We have included 20 SNPs and two deletion belonging to twelve different genes (Table 1, Table S3). Some of the associations have been investigated from a functional perspective^4^ while others have only been recently detected on GWAS studies and their potential role in the protection against the disease is not yet well understood.

*G6PD*, the key enzyme in the oxidative pentose phosphate pathway is probably one of the most prevalent signs of malaria-resistance in European populations, at least in the Mediterranean area. The *G6PD* A- is the most prevalent resistant allele in African populations. The B- mutation, known as Mediterranean *G6PD* has a more severe effect and is commonly found around the Mediterranean islands, in places where malaria was endemic, in frequencies of 2–20%. We have analysed the SNPs associated to the *G6PD* A- (rs1050828, rs1050829) and Mediterranean *G6PD* (rs5030868) forms. Additional *G6PD* mutations associated to malaria resistance have also been included (rs137852314, rs76723693, rs137852328)^43^.

Haemoglobin beta locus (*HBB*) determines the sequence of two types of polypeptidic chains in adult haemoglobin, HbA. Variants in the *HBB* gene such as HbC and HbS change the structure of the erythrocytes and confer resistance to *P. falciparum* in Asian and African populations^44^. The mutations that cause both HbS (rs334) and HbC (rs33930165) were screened^1, 45^. Recent studies based on GWAS have found an additional mutation in the *HBB* gene related to malaria resistance (rs372091)^12^. HbE caused by rs33950507 mutation) is a mainly Asiatic variant of *HBB* gene that also confers resistance to the infection of *P. falciparum* ^46^.

Duffy blood group system, Fy (a-b-) (*ACKR1* gene) confers resistance to *P. vivax* and *P. knowlesi* through the mutation rs2814778 that changes the structure of the protein, which is a receptor for *Plasmodium* in the erythrocytes^47^. This phenotype is especially present in West African populations were *P. vivax* originated^13^.

Additional mutations have been detected by GWAS analysis at *MARVELD3* (rs2334880), *ATP2B4* (rs4951074 and rs10900585) and *ABO* (rs81767199)^12^. The *MARVELD3* gene encodes for a tight junction-associated transmembrane protein of endothelial cells, a key point in the pathology of malaria infection. This mutation is supposed to confer resistance to *P. falciparum* malaria although it is not confirmed^12^. The *ABO* gene encodes for a glycosyltransferase enzyme. Mutations in this enzyme determine the ABO blood group. The individuals who are homozygous for a single nucleotide deletion (rs8176719) are classified as blood group O^48^. O blood group is linked with a reduced risk of severe malaria^28, 49^. However, we note that, besides malaria, the O blood group has been associated to susceptibility to other pathogens such as *Helicobacter pylori* ^50^ and severe cholera^51^. We have also analyzed the rs8176746 SNP. This mutation, which is in LD with the rs8176719 variant, determines the production of B antigens^48^. The presence of the derived allele in this locus is associated with an increased risk of severe malaria^28^. Evidence of malaria protection has been also described at *CD40LG* gene in Gambian populations: homozygotes for the derived rs3092945 allele show a reduced risk of suffering severe malaria. However, a significant increase of severe malaria risk was found for the same allele in Kenyan populations^28^. In the same study, a severe malaria protective effect of the SNP rs201346212 heterozygous genotype (*CD36* gene) was described. Other possible evidences on *P. falciparum* malaria resistance include additional genes such as *GRK5*, a G protein receptor (mutation rs2230345)^52^, *IL-10* (rs1800890), *FCGR2B* (rs105050) and *TIRAP* (rs8177374).

### Imputation

Due to the low coverage in many ancient samples and gaps associated to the targeted capture data, we performed imputations of SNPs not covered by any DNA read using Beagle-4 software^14^ and the Tuscans (TSI) population of the 1,000 Genomes project^53^ as a reference panel.

Some of the alleles screened involve C to T or G to A changes that could be affected by *post-mortem* DNA degradation^54^. To control for this confounding factor we have screened the genetic background in which the SNP is located, following the same approach as in ref. 9. In brief, the problematic SNP was removed on each ancient genome, genotype likelihoods were estimated and the SNP was imputed using Beagle-4 and the phased information of the 1,000 Genomes. This approach provides us with a probability estimate that a specific allele in a given sample is likely to be there or not, even in the absence of reads covering that nucleotide position. We have considered genotypes that yielded a probability higher than 0.95.

The genotypes obtained from genome sequence data and validated with Beagle-4 software were used to generate genotype frequencies. The allelic frequencies were estimated using both validated genome sequence data and targeted capture data. Because of the impossibility to infer genotypes in the case of targeted capture data only one random allele per position was taken into account.

### Statistical analysis

The allelic frequencies obtained with validated genotypes and targeted capture data were stratified by geographic position of the samples (North Europe and South Europe -the latter including Iberia, Italy, the Balkan peninsula and Anatolia-) and period (Upper Palaeolithic, Early Neolithic, Middle Neolithic, Late Neolithic, Bronze Age, Iron Age, Roman Age, Post-Roman Age).

To explore potential differences in the genotypic composition between modern South (Iberians and Tuscans) and North Europeans (Finnish, British and CEU) populations, we tested two different genetic models (codominant and logistic additive models) for each SNP, with a case-control approach. A likelihood ratio test was estimated for each SNP and both models using a Bonferroni correction. A Fisher’s exact test with a case-control approach was used for comparing allele frequencies in ancient versus modern European populations in those SNPs with more than one detected case of derived allele. Due to the assumption that ancient frequencies are directly comparable to the modern ones, the Fisher’s exact test is anti-conservative here; however we have included the p-value in the cases were we found an>1 OR, just as an additional, supporting evidence.

### Population structure analysis

To determine the level of differentiation and substructure among subpopulations the statistic FST^55^ was calculated. The fixation index was determined performing all the possible pairwise calculations comparing North-South present-day Europeans, the five European populations of 1000 genomes, North-South ancient Europeans and Period-stratified ancient Europeans (the populations were clustered in three supra populations: Upper Paleolithic; Pre-Neolithic, Middle-Neolithic, Past-Neolithic; Bronze Age, Iron Age, Roman, Post-Roman). Nine SNPs that exhibited variability (Table 2) were used to perform the comparisons. To test the divergence and the stratification within the population the statistic FSI (where I stands for individual and S for sub-population), was also used to estimate the departure from panmixia at the sub-population level.

Additionally the G-test was calculated as it is described in Goudet *et al*.^56^. The authors showed that the likelihood ratio G-test, is the most powerful statistic to detect population differentiation, particularly when samples are unbalanced. These authors also showed that for diploids, the appropriate unit to randomize is the diploid genotype rather than the allele (of course, the contingency tables are based on alleles, not genotypes). The likelihood ratio G-statistic was performed on a contingency table of alleles at one locus x sampling unit; the individual genotypes were randomly permutated among samples and the G-statistic obtained from the original dataset is compared to the G-statistics obtained from the permuted datasets, yielding a P-value of the test.

### Neutral selection testing

To test for the presence of selection driving the genotypic frequencies in past-populations and reject neutrality we used a specific software (https://github.com/Schraiber/selection) as described in ref. 15. This analysis based on Bayesian probabilities was performed for each allele that presented diversity among ancient individuals (Fig. 3). The 92 ancient samples with complete genotypic information were grouped in eight periods (Upper Palaeolithic, Early Neolithic, Middle Neolithic, Late Neolithic, Bronze Age, Iron Age, Roman and Post-Roman) to create a time series. The number of derived alleles was counted in each period, and selection coefficients were inferred from allele frequencies along time. The analysis was performed assuming a constant population size and 10,000 individuals of effective population size. The dates used in the simulations were: −0.04, −0.018, −0.015, −0.008, −0.006, −0.004, −0.0004 and 0 relatively to the most recent analysed age (post-Roman period). Two SNPs (rs951074 and rs2334880) presented high frequencies of the derived allele in all the sampling times. Because of the software models the allele arising from a new mutation, a new mutation is much more likely to rise to high frequency if it is under selection. In the analysis of rs951074 and rs2334880 SNPs the origin of the derived allele was not modelled because it was already extended in the initial periods. The value distributions of selection coefficients over time were plotted with R 3.2.2 (http://cran.r-project.org) software.

## Electronic supplementary material

Supplementary Dataset 1

Supplementary Figures
