# Supplementary material for: Malaria was a weak selective force in ancient Europeans
Source: Sci Rep. 2017 May 3;7:1377. doi: 10.1038/s41598-017-01534-5 (PMC5431260; doi:10.1038/s41598-017-01534-5)
Supplement: Supplementary file 2 — Supplementary Figures [file 41598_2017_1534_MOESM2_ESM.pdf]

Supplementary Figures S1-S8

Malaria was a weak selective force in ancient Europeans

Pere Gelabert[1], Iñigo Olalde[1],Toni de-Dios[1], Sergi Civit[2], Carles Lalueza-Fox[1]

[1]Institute of Evolutionary Biology (CSIC-Universitat Pompeu Fabra), 08003 Barcelona (Spain)
[2]Department of Statistics, Faculty of Biology, University of Barcelona, 08028 Barcelona (Spain)

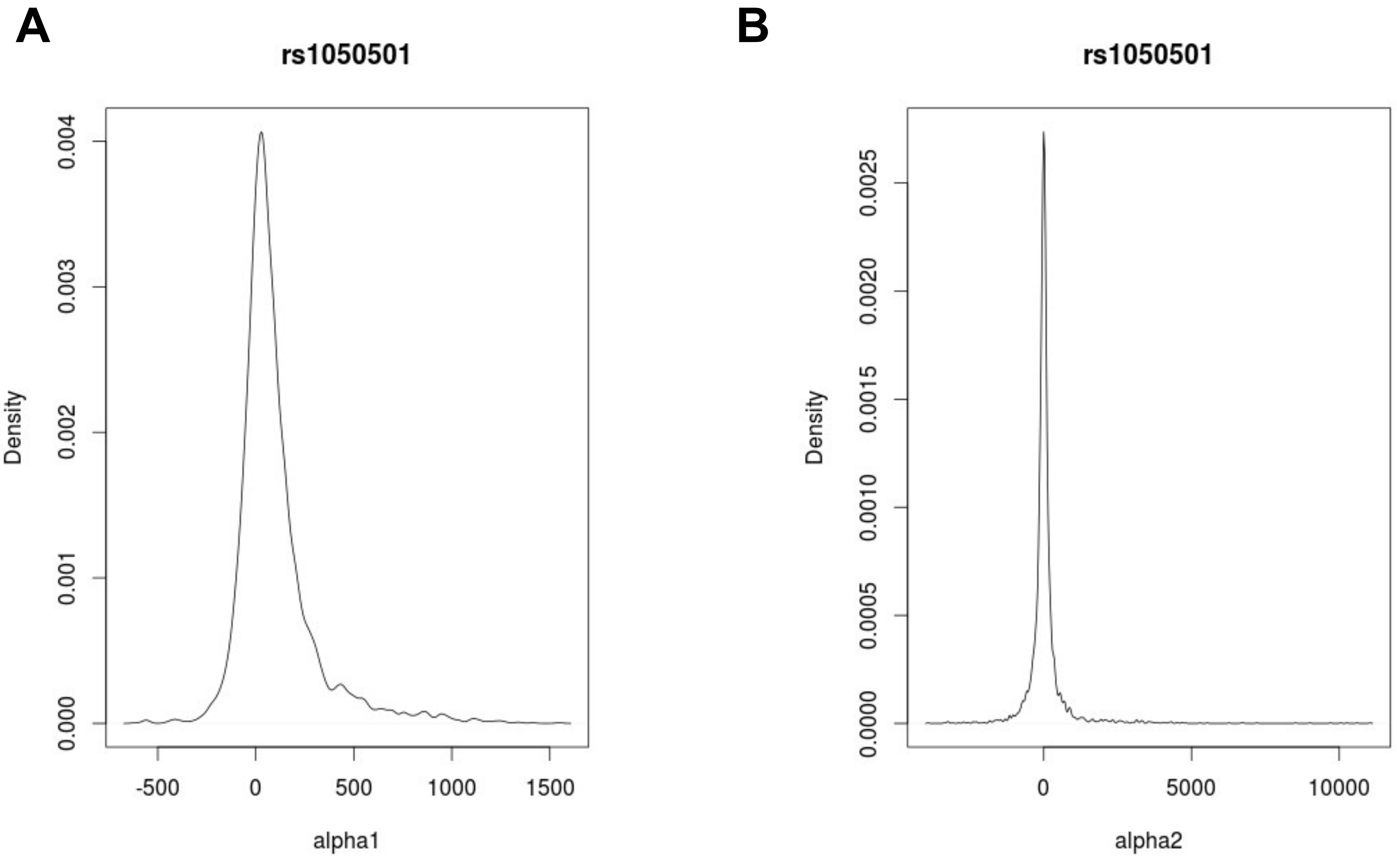

Figure S6: Posterior distributions of selection coefficients alpha 1(A) and alpha 2(B) for the rs1050501 locus.

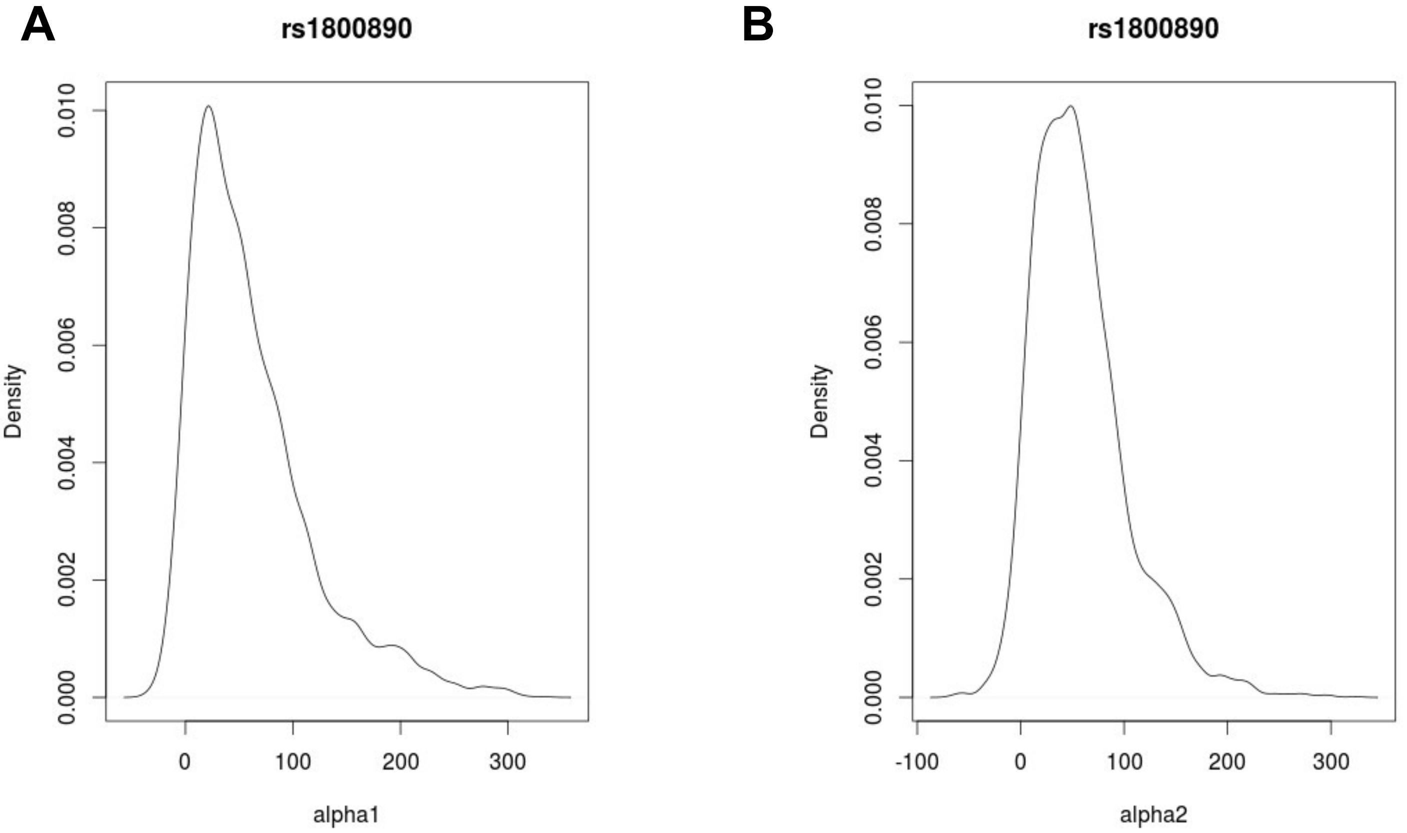

Figure S7: Posterior distributions of selection coefficients alpha 1(A) and alpha 2(B) for the rs1800890 locus.

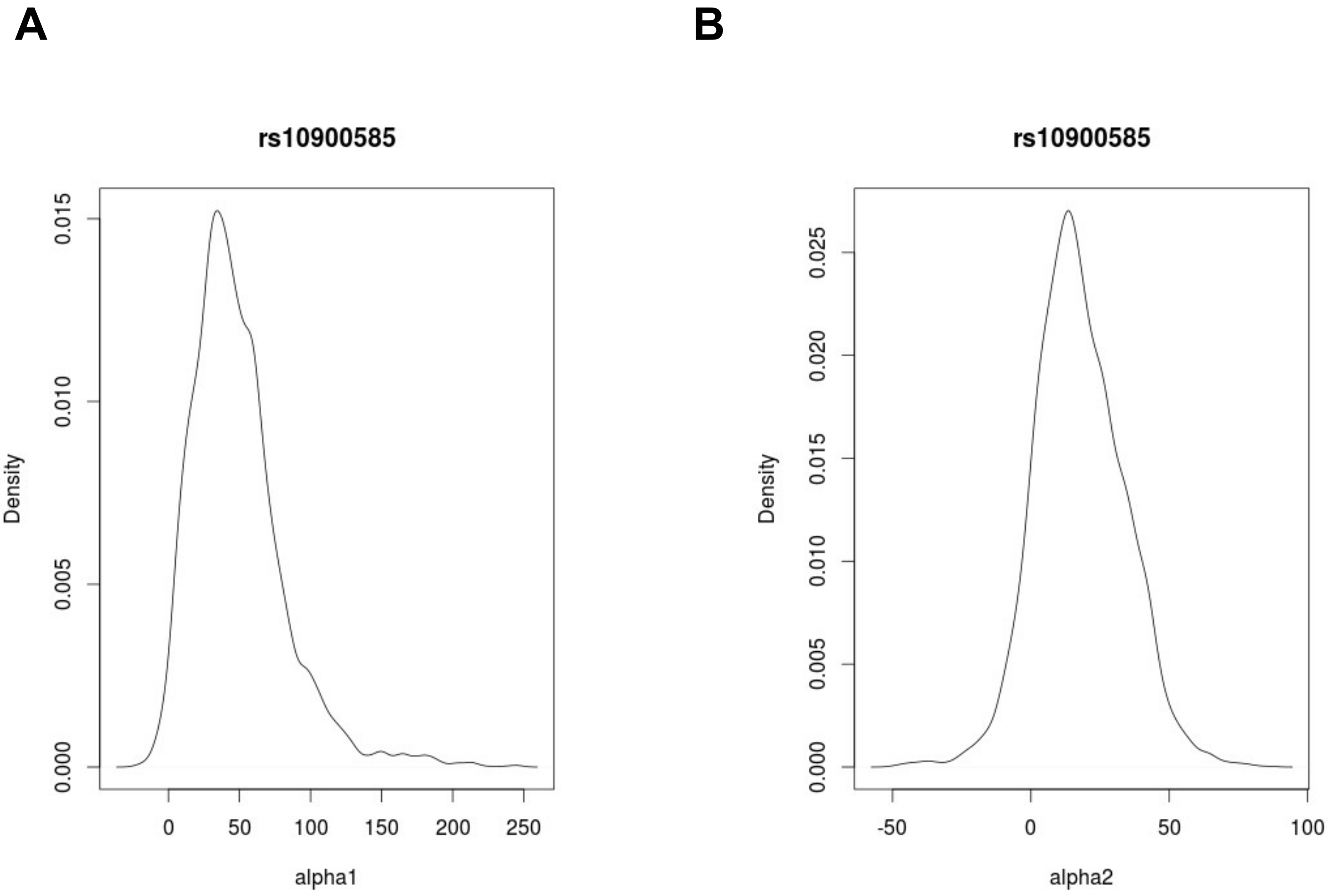

Figure S8: Posterior distributions of selection coefficients alpha 1(A) and alpha 2(B) for the rs10900585 locus.

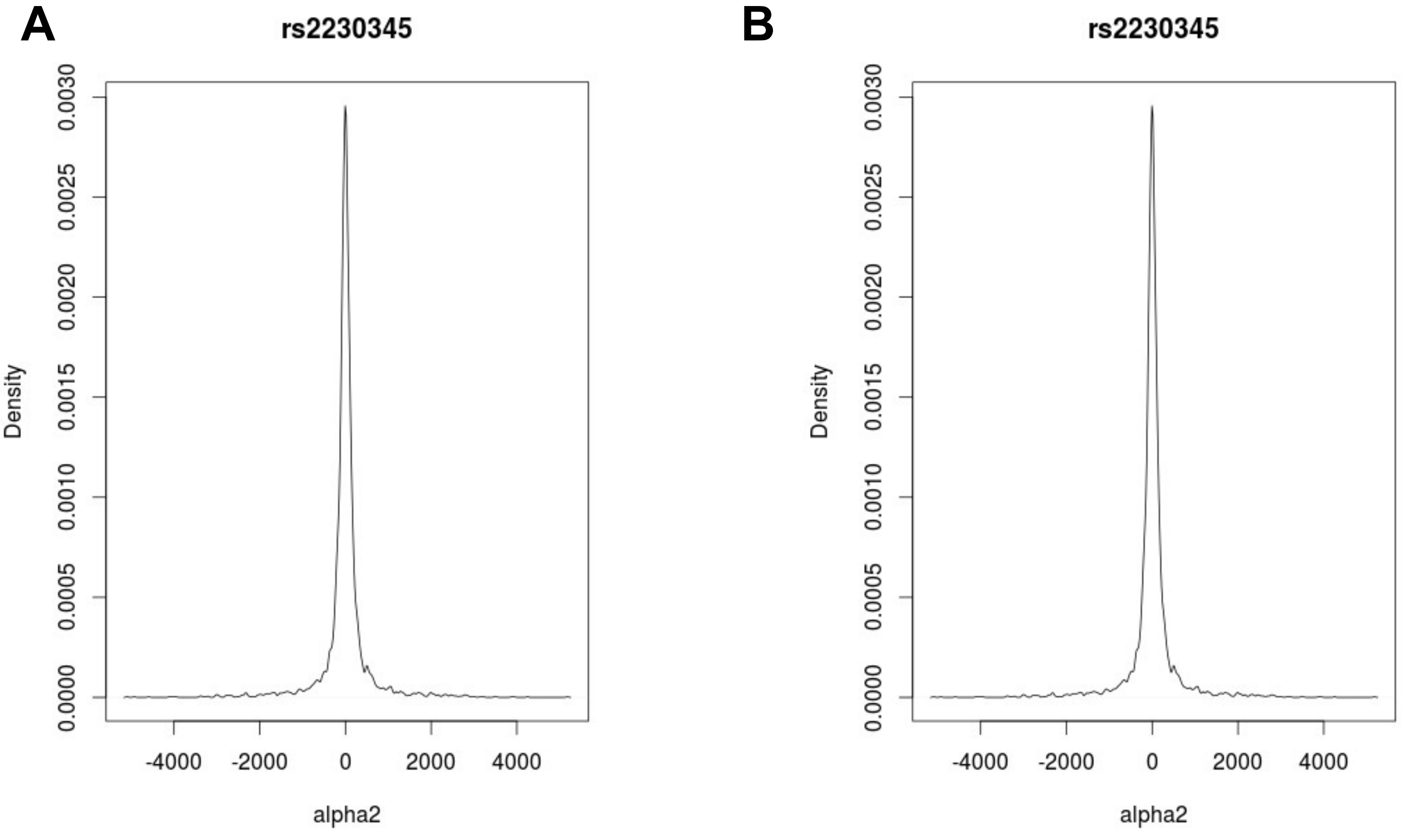

Figure S9: Posterior distributions of selection coefficients alpha 1(A) and alpha 2(B) for the rs2230345 locus.

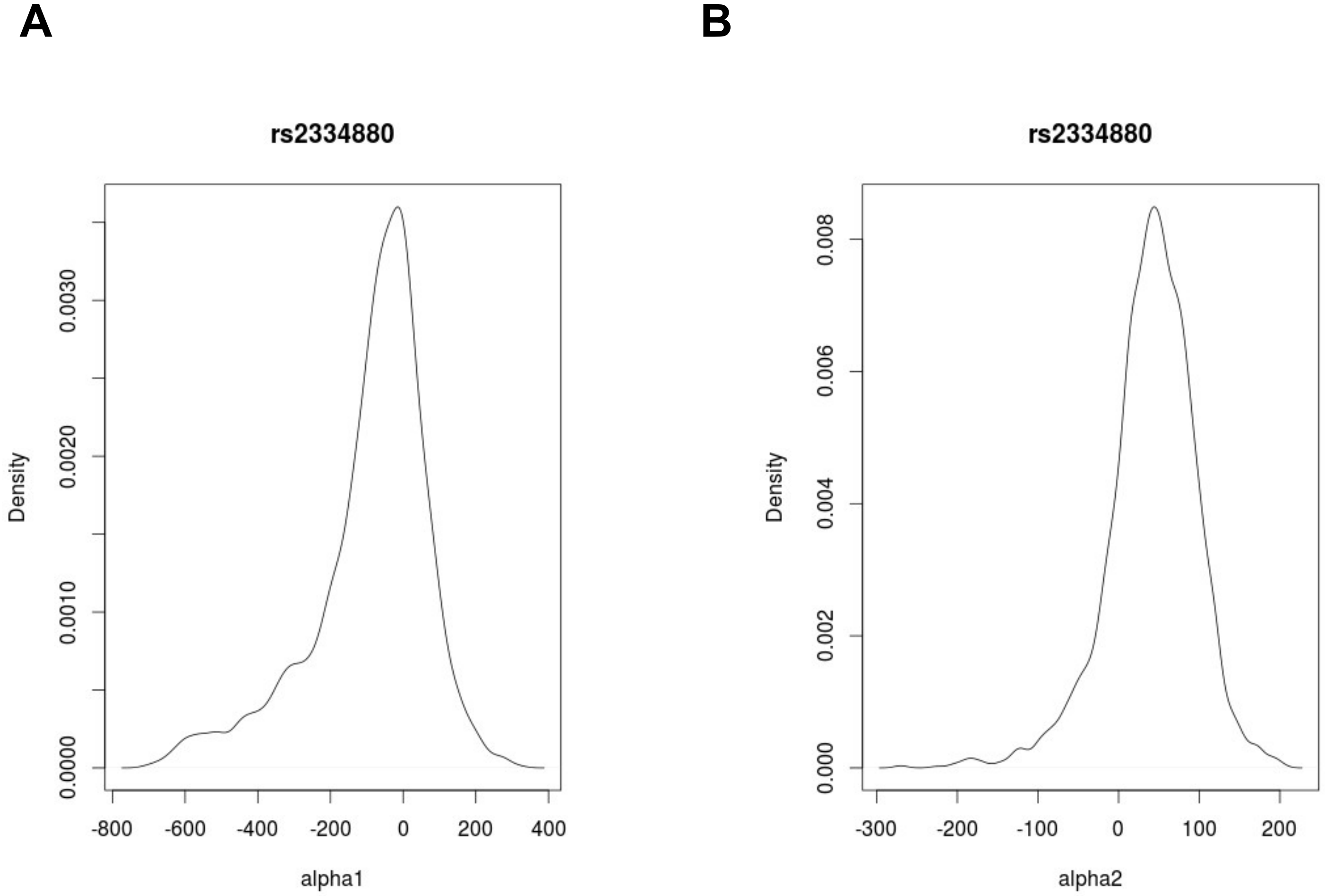

Figure S10: Posterior distributions of selection coefficients alpha 1(A) and alpha 2(B) for the rs2334880 locus.

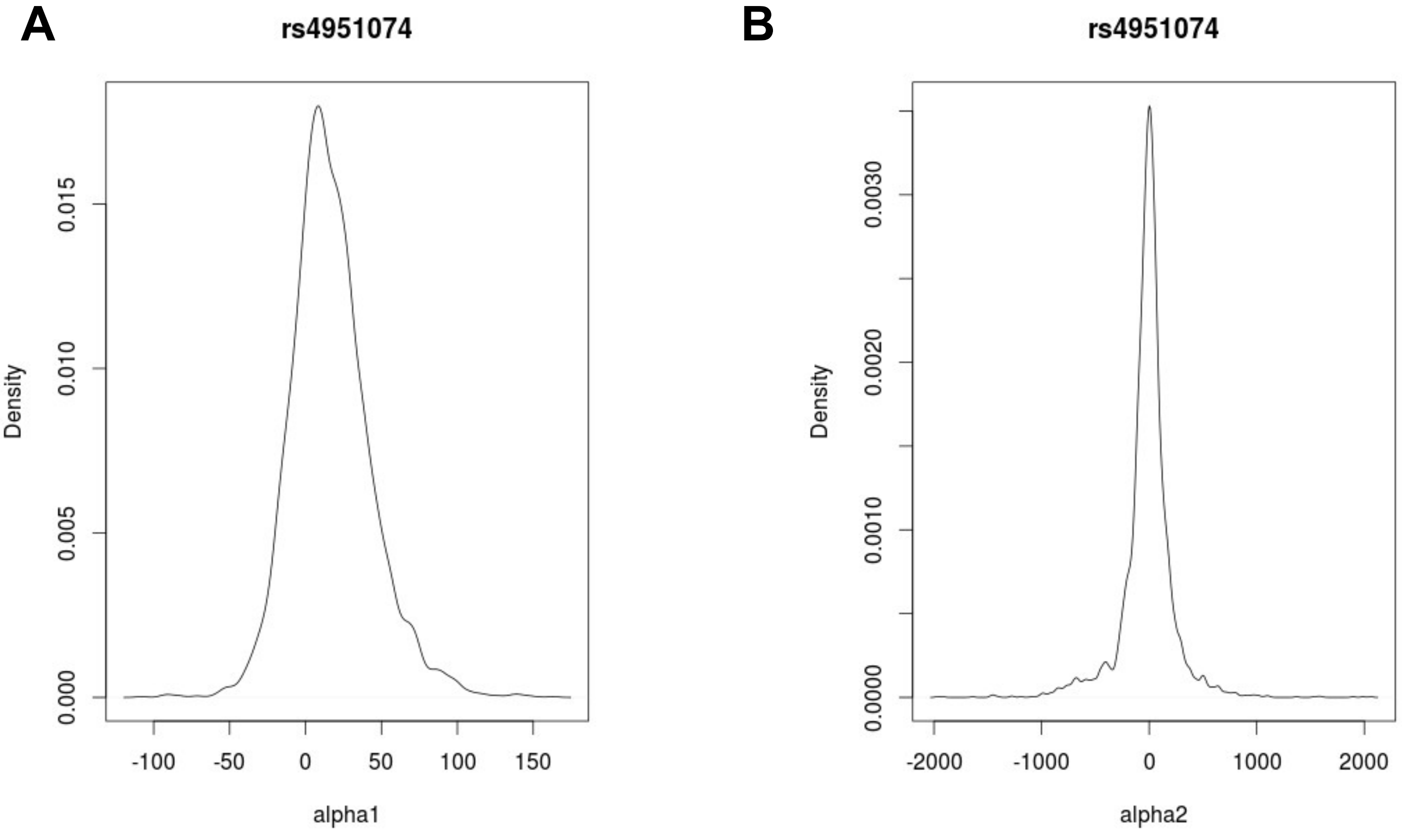

Figure S11: Posterior distributions of selection coefficients alpha 1(A) and alpha 2(B) for the rs4951074 locus.

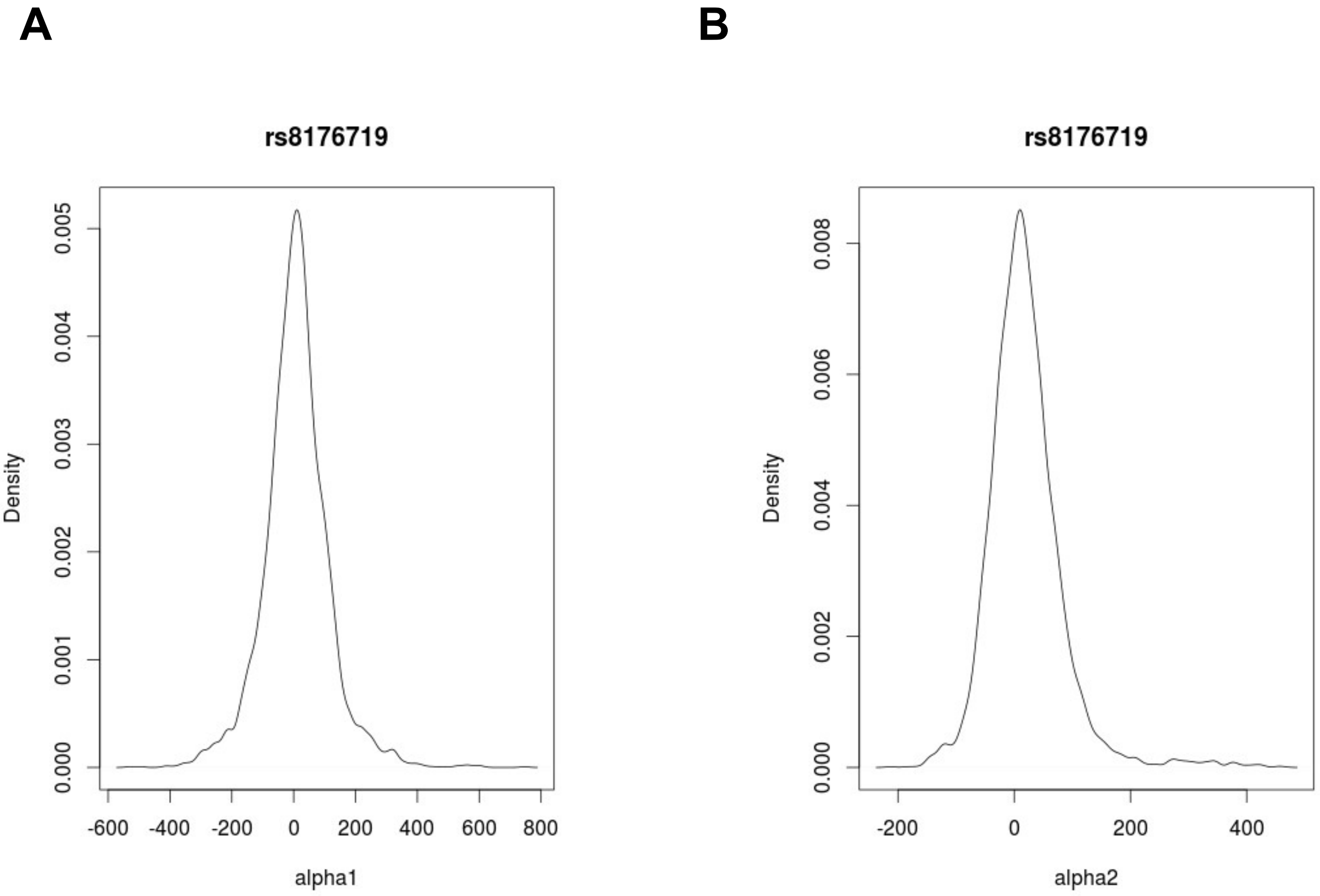

Figure S12: Posterior distributions of selection coefficients alpha 1(A) and alpha 2(B) for the rs8176719 locus.

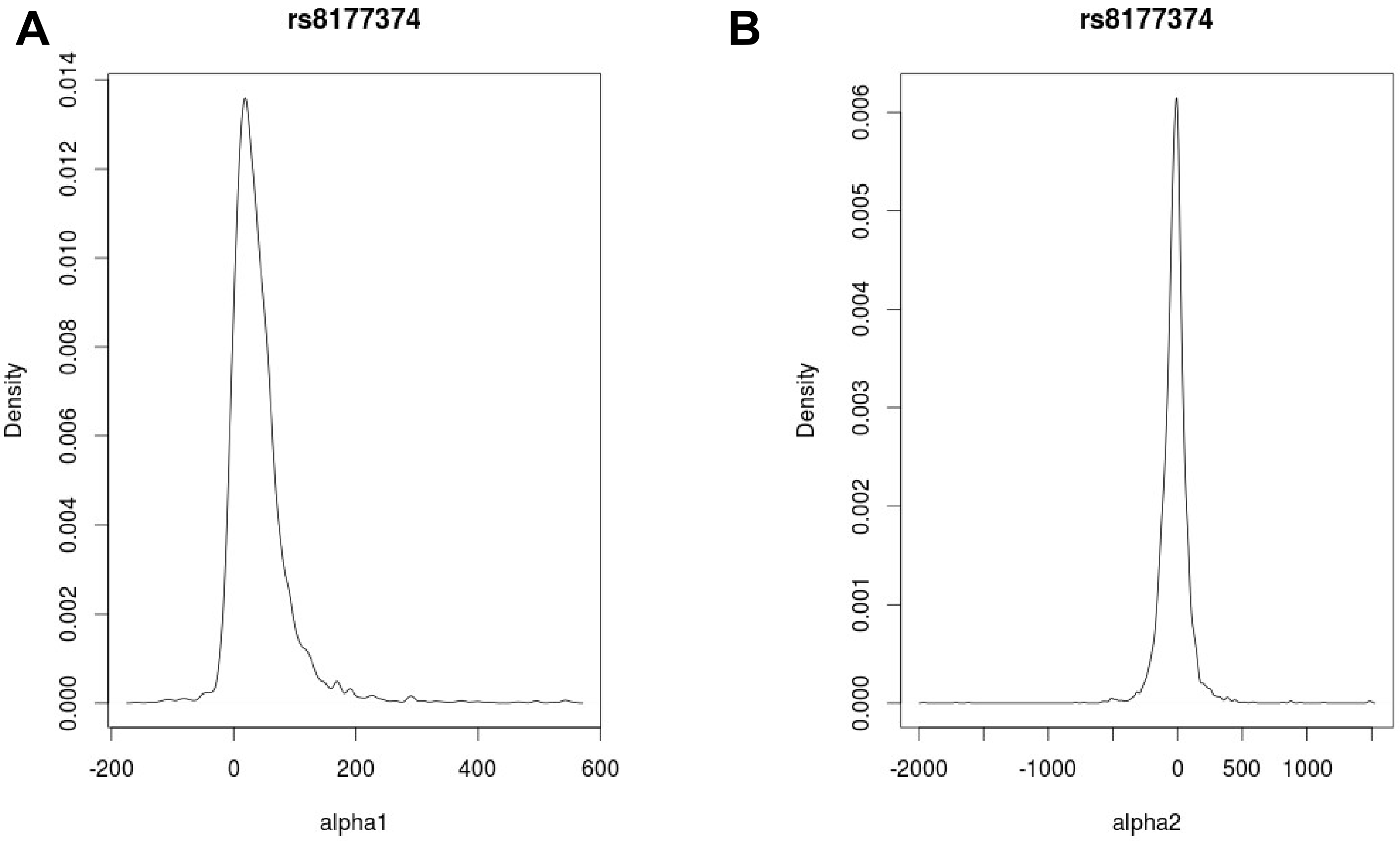

Figure S13: Posterior distributions of selection coefficients alpha 1(A) and alpha 2(B) for the rs8177374 locus.
